# Supplementary material for: Prehospital Management of Postpartum Hemorrhage—A National, Cross-Sectional Study in Norway
Source: Healthcare (Basel). 2024 Sep 21;12(18):1894. doi: 10.3390/healthcare12181894 (PMC11431836; doi:10.3390/healthcare12181894)
Supplement: Supplementary file 1 [file healthcare-12-01894-s001.zip › healthcare-3204778-supplementary.pdf]

Knowledge and competence in prehospital post-partum handling

Questions marked red not included in the current study

Background

1. **What is your educational background? (several options possible)**

Ambulance- assistant	
Ambulance worker (Upper High School)	
Bachelor paramedicine/paramedic	
Bachelor nursing	
Paramedic further education	
Other (please write with your own words)	

2. Years of experience from current workplace? (number of years)

3. Years of experience from working in ambulance, in total? (number of years)

4. Is your employment permanent?

Yes	No	Part time (% position)	On-call

5. Gender: Male Female

6. Age (number of years)

7. Where do you work (which ambulance station)?

Knowledge

8. How much is normal hemorrhage during birth, and when is it defined as postpartum hemorrhage? (please describe in your own words)

9. Which interventions should be initiated in postpartum hemorrhage? (please describe in your own words)

10. How do you estimate the amount of hemorrhage during/after birth? (please describe in your own words)

11. When you observe a life threathening hemorrhage, what do you do first? (please describe in your own words).

12. Are there other clinical situations than postpartum hemorrhage where manual aortic compression can be lifesaving? (please describe in your own words)

13. Do you have any drugs for use in situations of postpartum hemorrhage in the ambulance?

Yes No Undecided

If yes, which ones? (please describe in your own words)

What are the side-effects of these drugs? (please describe in your own words)

14. When is manual aortic compression appropriate? (please describe in your own words)

15. When is manual aortic compression not appropriate? (please describe in your own words)

16. How would you provide manual aortic compression? (please describe in your own words)

17. What is the intention with manual aortic compression? (please describe in your own words)

18. How do you assess whether the manouvre is conducted correct? (please describe in your own words)

19. Which considerations do you do regarding drug administration during manual aortic compression? (please describe in your own words)

20. Are there any complications related to manual aortic compression?

Yes No Undecided

If yes, which complication? (please describe in your own words)

Added question:

Which procedure(s) is/are used in your ambulance station in postpartum hemorrhage?

External aortic compression Bimanual uterus compression Other None of the above

If other, please elaborate (freetext):

Knowledge- and competence needs

Added question:

Have you received any education or training in postpartum hemorrhage?

Practical simulation   Theoretical education Read a procedure   Other   No

21. Do you want more education in handling postpartum hemorrhage?

**Yes**	**No**	**Undecided**
☐	☐	☐

22. Do you want more training/simulation in handeling postpartum hemorrhage?

**Yes**	**No**	**Undecided**
☐	☐	☐

Experience

23. Do you have any experience(s) with postpartum hemorrhage?

Yes	No	Undecided
☐	☐	☐

24. Have you ever used manual aortic compression on a patient?

Yes   No   Undecided

☐ ☐ ☐

25. Have you ever considered using manual aortic compression on a patient?

Yes No Undecided

☐ ☐ ☐

26. Have you ever had patients with massive hemorrhage where you in retrospect see that you could have used manual aortic compression?

Yes **No** **Undecided**

☐ ☐ ☐

27. What was the main reason for not conducting manual aortic compression?

Lack of education Lack of training Uncertainty Difficult to cause pain/discomfort
